# Esophageal Inlet Patch

**DOI:** 10.1155/2011/460890

**Published:** 2011-03-30

**Authors:** C. Behrens, Peggy P. W. Yen

**Affiliations:** ^1^Radiology Residency Program, University of British Columbia, Vancouver, BC, Canada V6T 1Z4; ^2^Radiology Residency Program, Dalhousie University, Halifax, NS, Canada B3H 4R2

## Abstract

An inlet patch is a congenital anomaly consisting of ectopic gastric mucosa at or just distal to the upper esophageal sphincter. Most inlet patches are largely asymptomatic, but in problematic cases complications related to acid secretion such as esophagitis, ulcer, web and stricture may occur. The diagnosis of inlet patch is strongly suggested on barium swallow where the most common pattern consists of two small indentations on the wall of the esophagus. The diagnosis of inlet patch is confirmed via endoscopy with biopsy. At endoscopy, the lesion appears salmon-coloured and velvety and is easily distinguished from the normal grey-white squamous epithelium of the esophagus. The prominent margins correlate with the radiological findings of indentations and rim-like shadows on barium swallow. Histopathology provides the definitive diagnosis by demonstrating gastric mucosa adjacent to normal esophageal mucosa. No treatment is required for asymptomatic inlet patches. Symptomatic cases are treated with proton pump inhibitors to relieve symptoms related to acid secretion. Strictures and webs are treated with serial dilatation and should be biopsied to rule out malignancy.

## 1. Clinical Presentation

A 65-year-old male was referred to radiology for a barium meal study with complaints of high dysphagia for solids with occasional choking and regurgitation. Chest radiography and electrocardiogram examinations were normal. The barium study was performed with rapid sequence filming of the pharynx during swallowing at 6 frames per second in anteroposterior (AP) and lateral projections. Because of the characteristic radiological findings, endoscopy was arranged the following day to confirm the diagnosis.

## 2. Diagnosis

Esophageal inlet patch (also called cervical inlet patch, ectopic or heterotopic gastric mucosa of the upper esophagus).

## 3. Radiologic Findings

**Figure 1:**
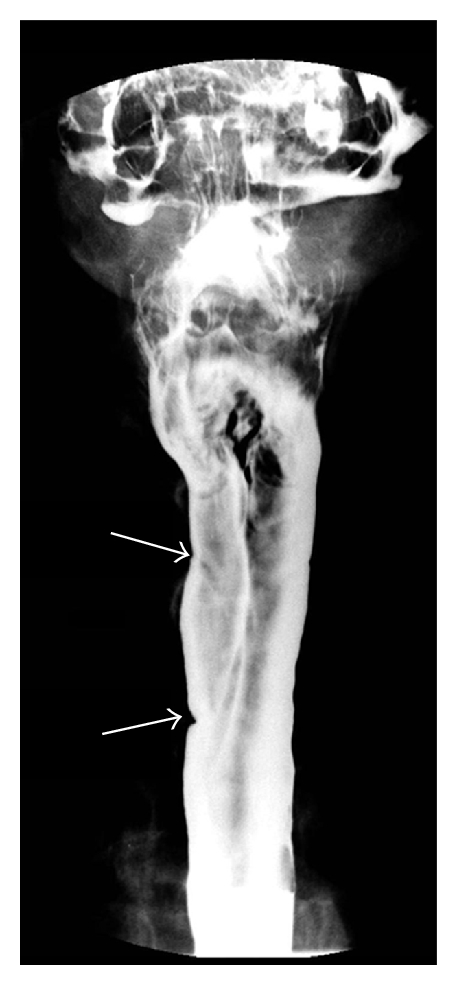
AP view of barium swallow showing two indentations (arrows) above the thoracic inlet and a slight lateral bulge of the esophageal lumen between the indentations.

**Figure 2:**
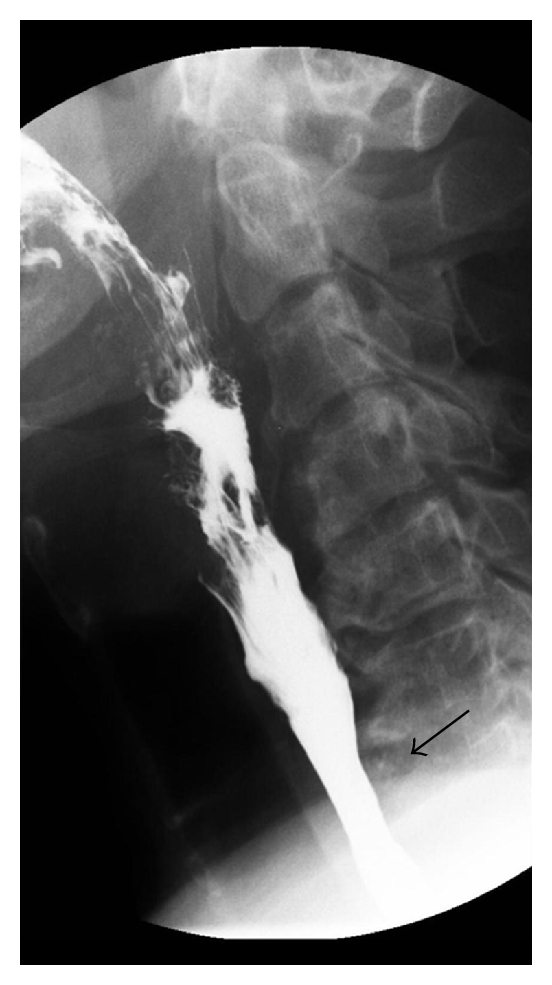
Lateral view of barium swallow showing a mild narrowing of the esophageal lumen (arrow) at the level of the thoracic inlet.

The barium swallow in the AP view taken at full cervical distension demonstrated two indentations in the barium column on the right (Figure 1, arrows) above the thoracic inlet. In between these indentations the barium column is bulging slightly outwards. These findings are characteristic of an esophageal inlet patch [1, 2]. On the lateral view, there is slight narrowing of the barium column at the thoracic inlet (Figure 2, arrow). The narrowing represents an esophageal stricture that is likely secondary to acid secretion by the inlet patch and is contributing to the patient's dysphagia.

## 4. Discussion

Ectopic gastric mucosa can occur anywhere along the gastrointestinal (GI) tract. When it occurs in the upper esophagus, it is called “inlet patch” because of its location at or just distal to the upper esophageal sphincter. The inlet patch is considered a congenital anomaly found in 10% of the population with careful searching at endoscopy [3] but it is often overlooked by endoscopists and radiologists and studies frequently report a prevalence between 0.1 and 3% [1, 4–6]. Inlet patches are believed to be due to incomplete transformation from columnar to squamous epithelium during embryonic development [5]. Squamous transformation starts in the mid-esophagus and extends bidirectionally and incomplete terminal transformation at the proximal esophagus accounts for the postcricoid location of inlet patches [7]. Most inlet patches are solitary and extend longitudinally, affecting only part of the circumference, but some are annular and multiple lesions are not uncommon [1, 3, 5].

Most inlet patches are largely asymptomatic, but in problematic cases complications related to acid secretion such as esophagitis, ulcer, web, and stricture may produce symptoms such as chest and throat pain, dysphagia, globus sensation, and shortness of breath [6–8]. The size of the patch is related with dysphagia severity, possibly as a function of increased acid secretion [9]. In some cases of inlet patch ulcer, serious and life-threatening sequelae such as hemorrhage, perforation, and tracheoesophageal fistula may occur [6]. Amongst those with concurrent inlet patch and gastric *H. pylori*, 73% will have an infected inlet patch [6] which may exacerbate complications and related symptoms. Chronic cough and hoarseness have been reported in association with inlet patches, presumably due to acid irritation of the airways and vocal cords [6, 10]. Adenocarcinoma may arise in the ectopic gastric mucosa but this is rare and is considered sporadic. In contrast to Barrett's esophagus there is no increased risk for adenocarcinoma associated with inlet patches as they are not metaplastic [6].

The diagnosis of inlet patch is strongly suggested by characteristic findings on barium swallow [1, 2]. The lesions are most evident when the cervical esophagus is at maximum distension following the opening of the upper esophageal sphincter. Characteristic findings are discussed in [1, 2]. The most common pattern consists of two small indentations on the wall of the esophagus. Alternatively the indentations may be more prominent with an intervening bulge away from the esophageal lumen, as was noted in the images of the case presented here or there may be only a single indentation. Other possible findings reflect the prominent border of the inlet patch and include rim-like shadows and irregular outlines.

**Figure 3:**
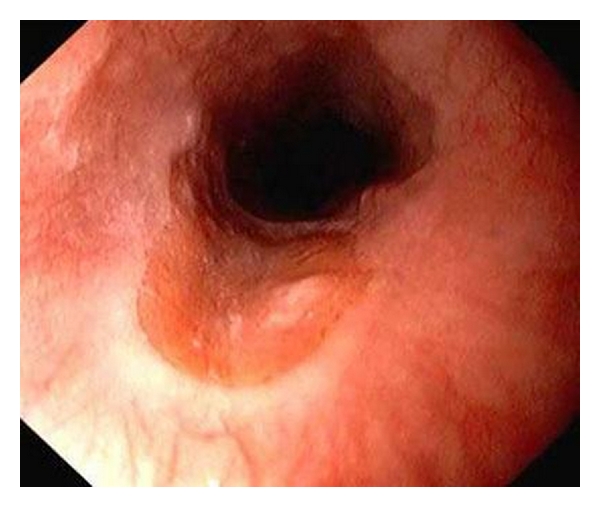
Endoscopic appearance of an annular inlet patch from a different patient. The ectopic gastric mucosa is salmon-coloured and velvety and is easily distinguished from the normal grey-white esophageal epithelium. The raised border of the lesion corresponds to the radiological findings of esophageal indentations. Image reprinted with permission from Medscape.com, 2009. Available at: http://www.medscape.com/viewarticle/405480.

**Figure 4:**
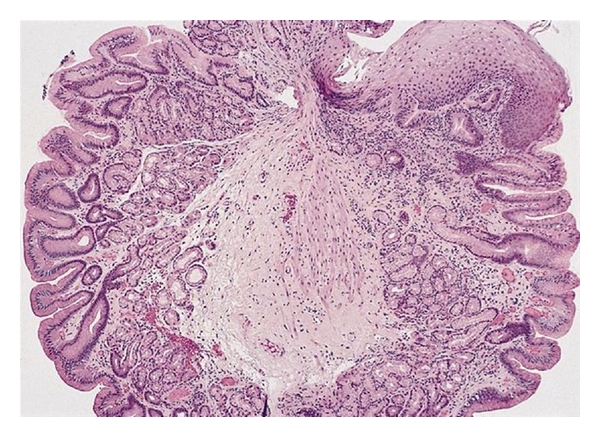
Histology of a portion of an inlet patch from a different patient showing gastric mucosa adjacent to normal esophageal mucosa with stratified squamous epithelium (top left). The gastric mucosa shown here is antral-type as there are no oxyntic (acid-secreting parietal cell) glands.

The diagnosis of inlet patch is confirmed via endoscopy with biopsy. The lesion will be seen more often by endoscopists whose custom is to withdraw the scope very slowly through the upper sphincter in order to inspect the arytenoids and vocal cords. At endoscopy, the lesion appears salmon-coloured and velvety and is easily distinguished from the normal grey-white squamous epithelium of the esophagus (Figure 3) [3, 6]. Inlet patches range from 0.2 to 5 cm and can be round or oval with a flat, slightly raised, or depressed surface and may have heaped margins most often on the lateral or posterior surfaces [3, 11]. The prominent margins correlate with the radiological findings of indentations and rim-like shadows on barium swallow. Histopathology provides the definitive diagnosis by demonstrating gastric mucosa adjacent to normal esophageal mucosa (Figure 4). Histopathological studies have demonstrated that oxyntic mucosa (gastric body-like with acid-secreting parietal cells) is the most common histologic subtype of esophageal ectopic gastric mucosa but cardiac, antral, and mixed types also occur [3, 4, 7].

A treatment strategy based on symptoms and underlying pathology is outlined in [6]. There is no treatment required for asymptomatic inlet patches. Affected individuals who are symptomatic may find relief with the use of proton pump inhibitors. Strictures and webs are treated with serial dilatation [4, 6] but should include biopsy to rule out malignancy [6]. Ablation of inlet patches has been shown to relieve globus [10] and has been used to successfully treat inlet patch dysplasia although its routine use in this context has not been determined [6].

As there was no evidence of mechanical obstruction, the cause of our patient's symptoms were thought to be secondary to esophageal irritation from acid-secretion. He responded well to treatment with a proton pump inhibitor.
